# The Surveillance for Enteric Fever in Asia Project (SEAP), Severe Typhoid Fever Surveillance in Africa (SETA), Surveillance of Enteric Fever in India (SEFI), and Strategic Typhoid Alliance Across Africa and Asia (STRATAA) Population-based Enteric Fever Studies: A Review of Methodological Similarities and Differences

**DOI:** 10.1093/cid/ciaa367

**Published:** 2020-07-29

**Authors:** Megan E Carey, William R MacWright, Justin Im, James E Meiring, Malick M Gibani, Se Eun Park, Ashley Longley, Hyon Jin Jeon, Caitlin Hemlock, Alexander T Yu, Abdramane Soura, Kristen Aiemjoy, Ellis Owusu-Dabo, Mekonnen Terferi, Sahidul Islam, Octavie Lunguya, Jan Jacobs, Melita Gordon, Christiane Dolecek, Stephen Baker, Virginia E Pitzer, Mohammad Tahir Yousafzai, Susan Tonks, John D Clemens, Kashmira Date, Firdausi Qadri, Robert S Heyderman, Samir K Saha, Buddha Basnyat, Iruka N Okeke, Farah N Qamar, Merryn Voysey, Stephen Luby, Gagandeep Kang, Jason Andrews, Andrew J Pollard, Jacob John, Denise Garrett, Florian Marks

**Affiliations:** 1 Department of Medicine, University of Cambridge, Cambridge, United Kingdom; 2 Public Health Surveillance Group, Princeton, New Jersey, USA; 3 International Vaccine Institute, SNU Research Park, Seoul, Republic of Korea; 4 Oxford Vaccine Group, University of Oxford, Oxford, United Kingdom; 5 Malawi-Liverpool-Wellcome Trust Clinical Research Programme, Blantyre, Malawi; 6 Department of Infectious Diseases, Imperial College London, London, United Kingdom; 7 Global Immunization Division, Centers for Disease Control Foundation, Atlanta, Georgia, USA; 8 Division of Epidemiology and Biostatistics, School of Public Health, University of California, Berkeley, California, USA; 9 Division of Infectious Diseases and Geographic Medicine, Department of Medicine, Stanford University, Stanford, California, USA; 10 Institut Supérieur des Sciences de la Population, University of Ouagadougou, Ouagadougou, Burkina Faso; 11 School of Medical Sciences, Kwame Nkrumah University of Science and Technology, Kumasi, Ghana; 12 Armauer Hansen Research Institute, Addis Ababa, Ethiopia; 13 Child Health Research Foundation, Department of Microbiology, Dhaka Shishu Hospital, Dhaka, Bangladesh; 14 Department of Microbiology, Institut National de Recherche Biomédicale, Kinshasa, Democratic Republic of Congo; 15 Department of Clinical Sciences, Institute of Tropical Medicine, Antwerp, Belgium; 16 Department of Microbiology and Immunology, KU Leuven, Leuven, Belgium; 17 Institute of Infection and Global Health, University of Liverpool, United Kingdom; 18 Centre for Tropical Medicine and Global Health, Nuffield Department of Medicine, University of Oxford, Oxford, United Kingdom; 19 Mahidol Oxford Tropical Medicine Research Unit, Mahidol University, Bangkok, Thailand; 20 Department of Epidemiology of Microbial Diseases, Yale School of Public Health, Yale University, New Haven, Connecticut, USA; 21 Department of Pediatrics and Child Health, Aga Khan University, Karachi, Pakistan; 22 International Centre for Diarrhoeal Disease Research, Bangladesh, Dhaka, Bangladesh; 23 Fielding School of Public Health, University of California, Los Angeles, Los Angeles, California, USA; 24 Division of Infection and Immunity, University College London, London, United Kingdom; 25 Oxford University Clinical Research Unit, Patan Academy of Health Sciences, Kathmandu, Nepal; 26 Department of Pharmaceutical Microbiology, Faculty of Pharmacy, University of Ibadan, Idaban, Nigeria; 27 Christian Medical College, Vellore, India; 28 Sabin Vaccine Institute, Washington, District of Columbia, USA

**Keywords:** blood culture, enteric fever surveillance, *Salmonella* Typhi, typhoid fever

## Abstract

Building on previous multicountry surveillance studies of typhoid and others salmonelloses such as the Diseases of the Most Impoverished program and the Typhoid Surveillance in Africa Project, several ongoing blood culture surveillance studies are generating important data about incidence, severity, transmission, and clinical features of invasive *Salmonella* infections in sub-Saharan Africa and South Asia. These studies are also characterizing drug resistance patterns in their respective study sites. Each study answers a different set of research questions and employs slightly different methodologies, and the geographies under surveillance differ in size, population density, physician practices, access to healthcare facilities, and access to microbiologically safe water and improved sanitation. These differences in part reflect the heterogeneity of the epidemiology of invasive salmonellosis globally, and thus enable generation of data that are useful to policymakers in decision-making for the introduction of typhoid conjugate vaccines (TCVs). Moreover, each study is evaluating the large-scale deployment of TCVs, and may ultimately be used to assess post-introduction vaccine impact. The data generated by these studies will also be used to refine global disease burden estimates. It is important to ensure that lessons learned from these studies not only inform vaccination policy, but also are incorporated into sustainable, low-cost, integrated vaccine-preventable disease surveillance systems.

Enteric fever, the collective term for typhoid and paratyphoid fevers, describes a systemic infection caused by *Salmonella enterica* serovars Typhi or Paratyphi A, B, or C. Recent estimates suggest that these organisms cause 14.3 million infections (95% confidence interval [CI], 12 500 000–16 300 000) and 136 000 deaths (95% CI, 77 000–219 000) annually [1]. Invasive nontyphoidal *Salmonella* (iNTS) disease is caused by other *Salmonella* serovars, most frequently by *Salmonella* Typhimurium, *Salmonella* Enteritidis, or *Salmonella* Dublin. Invasive nontyphoidal *Salmonella* disease caused an estimated 535 000 infections (95% CI, 409 000–705 000) and 77 500 deaths (95% CI, 46 400–123 000) in 2017 [2], of which 18 400 were attributed to human immunodeficiency virus. While improved water treatment and sanitation infrastructure have eliminated enteric fever as a public health problem in high-income countries, invasive *Salmonella* infections, which include iNTS, remain a public health issue in many low- and lower-middle-income countries.

A major impediment to understanding the true burden of enteric fever and iNTS disease is the lack of appropriately sensitive diagnostics and inconsistent usage of existing tests. Bone marrow culture is considered the gold standard for diagnosis of typhoid and paratyphoid fever, but given the invasive and challenging nature of obtaining bone marrow aspirate, it is rarely performed [3]. Often, treating physicians rely on a serological test like the Widal test, which has limited utility in endemic settings [4]. Blood culture–based diagnostics are recommended for use in surveillance of typhoid fever and other invasive *Salmonella* infections by the World Health Organization (WHO) [5], but these tests are not available in most low-resource settings, which often lack adequate resources and trained personnel required to conduct routine blood culture tests [3]; when they are available, blood cultures are only 40%–60% sensitive, depending in part on the volume of blood collected and prior antibiotic usage, and results are not available for several days, so are not useful for decisions on empiric therapy [6].

Often, febrile patients will not present to healthcare facilities for diagnosis and treatment. Potential deterrents to healthcare seeking include distance to and accessibility of the closest healthcare facility, or costs associated with treatment and/or hospitalization, combined with ease of access and affordability of antimicrobials in the community. As a result, the true number of invasive *Salmonella* infections may be underestimated.

In 2009, the WHO highlighted the need for additional data on the burden of invasive *Salmonella* disease [7]. At that time, early estimates of disease burden relied on extrapolation of data obtained from surveillance studies conducted in limited geographical regions, which did not entirely reflect the diversity of epidemiological settings in which typhoid is encountered [8]. A historic lack of population-based surveillance studies has also contributed to uncertainty around disease burden, particularly in the African continent. A review of the global burden of enteric fever conducted in 2004 showed that only 2 countries in Africa had conducted systematic, population-based surveillance between 1954 and 2000 (South Africa and Egypt) [8].

To address the limitations of existing data sets, several surveillance studies have been established over the past decade, funded primarily by the Bill & Melinda Gates Foundation and the Wellcome Trust. One of the first studies funded was the Typhoid Fever Surveillance in Africa Program (TSAP), coordinated by the International Vaccine Institute (IVI). The TSAP study demonstrated higher overall incidence rates of typhoid fever in sub-Saharan Africa than previously suspected across both rural and urban sites, as well as high incidence rates of iNTS disease across multiple sites [9]. In the years that followed, additional surveillance studies were funded to provide more data on the burden of disease in diverse epidemiological settings and to answer additional questions about clinical features of enteric fever, such as the prevalence of severe manifestations of disease and chronic intestinal carriage. These included the Severe Typhoid Fever Surveillance in Africa program (SETA, IVI); the Surveillance for Enteric Fever in Asia Project (SEAP, Sabin Vaccine Institute); the Strategic Typhoid Alliance Across Africa and Asia (STRATAA, University of Oxford); and the Surveillance of Enteric Fever in India (SEFI, Christian Medical College, Vellore). Preliminary data from these studies have helped to inform the WHO Strategic Advisory Group of Experts’ recommendation for typhoid conjugate vaccine (TCV) use in the control of typhoid fever in endemic settings [10], and additional data generated by these studies will help direct optimal use of TCVs going forward.

Each of these studies has been conducted across mutliple distinct epidemiological settings and aims to address subtly different questions relating to invasive *Salmonella* disease burden. In this article, we compare the methodological similarities and differences between these diverse and complementary studies. We also identify early lessons learned and outstanding data gaps, and issue recommendations for optimizing and sustaining surveillance systems going forward.

## STUDY SETTINGS AND METHODS

### Severe Typhoid Fever Surveillance in Africa (SETA)

The SETA program builds on the infrastructure established as part of the TSAP study to characterize the severity and long-term effects of typhoid fever and iNTS disease across Africa. TSAP collected blood culture data between 2010 and 2014 and generated typhoid incidence rates from sites in 10 sub-Saharan African countries: Burkina Faso, Ethiopia, Ghana, Guinea-Bissau, Kenya, Madagascar, Senegal, South Africa, Sudan, and Tanzania. The TSAP results showed a great deal of heterogeneity in typhoid incidence across sites, with crude rates ranging from 0 to 284 cases per 100 000 person-years, and that there is a high burden in both rural and urban sites [9]. SETA surveillance was continued at sites in Madagascar, Burkina Faso, and Ghana, and surveillance was extended to include additional sites in the Democratic Republic of Congo, Ethiopia, and Nigeria. These sites were selected based on existing evidence of typhoid transmission and clinical microbiology capacity, as well as to ensure geographical representativeness of key regions within the continent.

Within each of the SETA study areas, patients were recruited at healthcare facilities across multiple tiers. Each site included a referral hospital, which, to be included in SETA, had to be equipped with imaging and surgical capacity to identify and treat intestinal perforations, as well as primary or secondary healthcare centers, which enrolled less severe febrile subjects using broader enrollment criteria [11]. Each recruitment center was assigned a geographic catchment area from which a defined population was identified. In all countries apart from Madagascar, the catchment area of the primary and/or secondary centers was “nested” within the catchment area of the tertiary center. A healthcare utilization survey (HCUS) was conducted in households randomly selected from the nested and broader study catchment areas to estimate incidence rates based on the proportion of the population seeking healthcare at the respective study facilities.

Screening for study eligibility was systematically conducted at all study facilities, including inpatient, outpatient, surgical, and emergency wards at referral facilities. To augment case detection, *Salmonella* bacteremia detected in SETA laboratories from patients who were not enrolled in the study were also included, as well as patients with intestinal (ileal) perforation suspected to be due to typhoid, from referral hospitals. Intestinal perforation cases were recruited into the study regardless of whether or not the patient resided in the study catchment area. Upon enrollment, blood, stool, oropharyngeal, and urine samples were collected. Blood was subjected to conventional microbiological culture for detection and identification of bacterial pathogens as well as other immunological investigations; stool was cultured to assess acute carriage status, and urine was examined for antibiotic residues to determine patterns in antimicrobial pretreatment.

Patients with blood culture–confirmed *Salmonella* Typhi, *Salmonella* Paratyphi, and non-Typhi *Salmonella* serotype infections were recruited into the long-term follow-up component of the study. Two healthy household members and 4 healthy neighborhood controls were enrolled for each case, and the entire cohort was followed for 1 year with contact points at predefined intervals to collect clinical information, blood and stool samples, and cost-of-illness and quality-of-life assessments [11].

### Surveillance for Enteric Fever in Asia Project (SEAP)

SEAP is a multicountry, multisite, population-based surveillance study aimed at characterizing the burden of enteric fever in South Asia. The project had two phases: phase 1, a retrospective clinical record review of invasive *Salmonella* infections, and phase 2, a prospective surveillance study. Phase 1 showed that *Salmonella* Typhi and Paratyphoid A was isolated from 0.43% to 2% of blood cultures conducted at hospitals in Bangladesh, Nepal, Pakistan, and India [12].

The prospective component of the study—initiated in October 2016—was conducted at urban and periurban sites in Bangladesh, Nepal, and Pakistan. Sites were selected to represent diverse communities in South Asia, but choices were constrained by the availability of laboratories capable of performing high-quality blood cultures. In addition to patients meeting the inclusion criteria at the hospital sites, the SEAP study also recruited enteric fever cases from laboratory networks beyond the site hospitals. Blood specimens were collected for culture, and a subsample of participants provided urine samples to test for residual antibiotic metabolites to understand antibiotic usage patterns. Data were collected on clinical manifestations, markers of severity of illness, complications of illness, and antimicrobial resistance. In addition, in collaboration with the US Centers for Disease Control and Prevention, an economic study of enteric illness was implemented at all 3 sites [12].

SEAP used a hybrid surveillance approach, adapted from Luby et al [13, 14], combining facility-based surveillance with an HCUS, administered to a representative subset of households using a single-stage, cluster design; among patients with fever lasting 3 or more days, the proportion that sought care for the febrile episode at a study facility was used as an adjustment to the number of enteric fever cases at the facility, for the purpose of calculating incidence and disease severity rates. Geographic catchment areas were delineated at surveillance sites encompassing the majority of suspected enteric fever cases identified during phase 1. Catchment areas used administrative boundaries so that study staff could easily determine if patients resided in the catchment area [12]. SEAP used a hybrid surveillance approach, adapted from Luby et al [13, 14], combining facility-based surveillance with an HCUS, which was administered to a representative subset of households using a single-stage, cluster design [13]. The proportion within the surveyed group who reported a fever lasting 3 or more days and sought care for the febrile episode at a study facility was then used as an adjustment for care-seeking at the study facilities within the catchment area.

### Surveillance of Enteric Fever in India (SEFI)

There has been a lack of nationally representative enteric fever incidence data in India, as highlighted by a 2016 meta-analysis [15]. To fill these data gaps, the SEFI program was established by the Christian Medical College, Vellore, and its design was developed in collaboration with the Indian Council of Medical Research, the Translational Health Sciences and Technology Institute, the Indian Academy of Pediatrics, and other public health stakeholders [16]. The SEFI team established a 3-tiered surveillance system to estimate the age-specific incidence of enteric fever in children, examine the heterogeneity of incidence in diverse settings across India, and generate information on antimicrobial resistance patterns and cost of illness. Study enrollment began in October 2017.

The tier 1 surveillance for estimating the incidence of enteric fever in children consists of active, community-based surveillance in 1 rural and 3 urban sites, which were selected to be broadly representative of different geographic settings and population densities. At each of the 4 sites, 6000 children between 6 months and 15 years are followed each week for 2 years to estimate the age-specific incidence rates of enteric fever in children. At least 1 contact each month is in-person and the rest are either in-person or by telephone. Parents are also encouraged to reach out to the study team if a child has fever in between weekly follow-up points, and all participating families are given thermometers and diary cards. Any fever of 3 or more consecutive days is considered a suspected case, and the child is referred to a study facility on the fourth day of fever, where a blood culture is performed if the child has had fever in the past 12 hours. Risk factors and other demographic data are also collected from participating households to permit extrapolation of incidence estimates to other similar risk settings [17].

Tier 2 is passive, hospital-based surveillance, which has been harmonized across 6 secondary-care facilities in 6 settings (5 rural and 1 urban) with well-defined catchment populations between 100 000 and 400 000 each. All patients hospitalized at a study facility with a fever are considered eligible. Upon consent, a blood culture is collected irrespective of duration of fever or temperature. Those with blood culture–confirmed enteric fever are followed up for 28 days to capture costs and clinical complications. Biannual HCUSs are administered to 5000 randomly selected households in 100 clusters at each site. These are used to determine the probability of seeking healthcare at the study facility. The population denominators are estimated by projecting annual growth rates from the 2011 census for each village in the catchment area and validated by reviewing the population in the sampled clusters in the HCUS. The tier 2 surveillance system was designed to estimate the incidence of severe enteric fever (requiring hospitalization) across all age groups.

Tier 3 surveillance is being conducted at 8 laboratories linked to tertiary care hospitals. The aims of this component of the surveillance system are to generate estimates of the proportion of blood cultures that are positive for *Salmonella* Typhi or Paratyphi A and to characterize antimicrobial resistance patterns. Patients with nontraumatic ileal perforations are also enrolled in tier 2 and 3 surveillance and followed up for up to 90 days for clinical and health economic outcomes.

### Strategic Typhoid Alliance Across Africa and Asia (STRATAA)

The STRATAA consortium, which includes key partners from the University of Oxford’s Oxford Vaccine Group, the Malawi Liverpool Wellcome Trust Clinical Research Programme, the International Center for Diarrhoeal Disease Research, Bangladesh (icddr,b), and the Oxford University Clinical Research Unit (Vietnam and Nepal), has conducted a prospective multicomponent epidemiological study in 3 densely populated urban sites in Bangladesh, Nepal, and Malawi [18]. This study combines passive febrile illness surveillance with serological surveillance. A baseline population of approximately 100 000 was established at each site by demographic census. Field teams visited each individual house, recording GPS position and collecting epidemiological data at the household and individual level. A census update was conducted every 6 months at the Bangladeshi site and at 12 months in Nepal, and a final census was conducted at all 3 sites after 2 years. In addition, 2 HCUS and water, sanitation, and hygiene surveys were performed in each site during the surveillance period to generate additional data on healthcare-seeking behavior and to investigate potential disease risk factors and transmission routes.

Passive surveillance was conducted from June 2016 in referral hospitals and primary health centers. Patients living within the population catchment area who presented to a study facility reporting fever of 2 days or longer or having a documented temperature of ≥ 38°C were enrolled and consented and blood cultures were taken [18]. Age-stratified serosurveys were performed at each study site to assess seroincidence of typhoid infection. The resulting seroincidence rates will be used to validate and provide upper bounds on the incidence rates derived from blood culture surveillance, using host responses as a proxy for incidence of infection. Additionally, the serosurveys were designed to identify potential chronic carriers of *S.* Typhi. After identifying individuals with a high anti-Vi antibody response, follow-up with stool collection and culture was performed to identify stool shedding.

## DISCUSSION

### Study Similarities

There are important methodological similarities across these four studies. Each study includes passive, healthcare facility–based blood culture surveillance of febrile patients to generate crude age-stratified incidence rate estimates stratified by age. Each study team also conducts an HCUS and applies correction factors to these crude rates to account for the proportion of patients from within the catchment area or study population seeking care for febrile illness at a study facility. All 4 studies make an adjustment for eligible cases missed by the study (either patients who met eligibility criteria but were not approached for enrollment, or who were enrolled and chose not to consent, or both), as well as an adjustment for blood culture sensitivity. Antimicrobial resistance patterns from sites in all 4 studies are being analyzed, and whole-genome sequencing of selected *Salmonella* isolates will be available from all studies. Cost-of-illness studies are embedded within each of these studies, as is screening for chronic typhoid carriage.

The SEAP and SETA studies employ similar methods to calculate incidence, owing in part to efforts coordinated by the Scientific Advisory Process for Optimal Research on Typhoid Burden of Disease Project (SAPORT, Emory University) group to harmonize methods; therefore, the results are more easily compared. Both studies generate data on the incidence of severe disease and follow enteric fever patients for at least 6 weeks to characterize long-term sequelae. SEFI tier 2 surveillance processes also closely resemble that of SEAP and SETA, although enrollment criteria differ across all 3 studies.

Each of the studies contributes data to advance broader enteric fever control objectives. Epidemiological data generated from all 4 have been shared with policymakers in relevant countries in support of decision-making around TCV introduction. The impact of TCV deployment is being evaluated or will be evaluated at site(s) from 3 of the 4 studies, although the designs of these studies and the delivery strategies under evaluation differ. A TCV impact assessment is being conducted through Kharadar General Hospital in Karachi, Pakistan (SEAP), in response to an extensively drug-resistant typhoid outbreak [19]. The University of Maryland and University of Oxford with Typhoid Vaccine Acceleration Consortium (TyVAC) partners are conducting 3 large-scale TCV trials at STRATAA surveillance sites: a cluster-randomized efficacy trial in Bangladesh [20] and 2 individually randomized efficacy studies in Nepal and Malawi [21, 22]. Interim analysis of the Nepal RCT showed that TCV had an 82% vaccine efficacy at 1 year after vaccination in children < 15 years of age [23]. Leveraging SETA surveillance, the University of Cambridge and partners are planning 2 TCV studies through the THECA (Typhoid Conjugate Vaccine Introductions in Africa) consortium: a cluster-randomized trial in Ghana using a similar methodology to the TyVAC trial in Bangladesh, as well as a mass-vaccination campaign with cohort effectiveness evaluation in the Democratic Republic of Congo [24]. In addition, alternatives to blood culture surveillance—namely, seroepidemiology and/or environmental sampling—are being validated at sites in all 4 studies.

### Study Differences

There are several differences between the methodologies of these studies, which are important to consider when interpreting and comparing results. While there is some overlap, the objectives are not uniform across the 4 studies. Eligibility criteria differ, as do the type, number, and frequency of sample collections. The approach to estimating adjusted incidence rates differs between studies, and for SETA and SEFI, within study tiers as well. These and other general methodological differences are summarized in Table 1.

**Table 1.** Comparison of Surveillance Methods

	SETA	SEAP	SEFI	STRATAA
Design	Prospective passive, facility-based surveillance paired with population-based HCUS Prospective case-control cohort for long-term follow-up	Retrospective and prospective passive, facility-based surveillance paired with population-based HCUS	Tier 1: Prospective population-based cohort with active surveillance Tier 2: Prospective passive, hospital-based paired with population-based HCUS Tier 3: Laboratory-based surveillance	Prospective population-based cohort with passive surveillance paired with population-based HCUS and seroincidence surveys
Eligibility criteria	Primary/secondary health facilities • Objective fever of ≥ 38°C OR • Subjective fever ≥ 3 consecutive days in the last week • AND reside in the nested catchment area Referral hospitals • Subjective fever ≥ 3 consecutive days in the last week, OR • Clinically suspected typhoid fever • AND reside in the catchment area • OR pathognomonic gastrointestinal perforations even in the absence of laboratory confirmation and regardless of catchment area (special cases)	Outpatient • 3 days of consecutive fever in the last 7 days • AND reside in the study catchment area • AND physician must advise blood culture Inpatient • Clinical suspicion of enteric fever AND physician must advise blood culture OR • Confirmed diagnosis of enteric fever at any time during hospitalization OR • Nontraumatic ileal perforations, even in the absence of laboratory confirmation Laboratory: • Blood culture positive for *S.* Typhi or *S.* Paratyphi A only	Tier 1 • Subjective fever ≥ 3 consecutive days (families given thermometers and diary cards to record) • AND reside in census population area • AND fever in the last 12 hours before presentation, Tier 2 • All inpatients presenting with fever OR • Patient with nontraumatic ileal perforation • AND residing in geographic catchment area Tier 3 • Blood culture positive for *S.* Typhi or *S.* Paratyphi A only	• Objective fever of ≥ 38°C OR • Subjective fever of ≥ 2 days • AND reside in census population area
Sample collection and follow-up	• Blood samples taken from enrolled subjects at baseline • For blood culture–confirmed cases of *S.*Typhi and iNTS and associated controls, blood, urine, and stool samples and oropharyngeal swabs were taken at day 3-7, 14, 28, 90, 180, 270, and 360• Ileal tissue or other surgical samples taken in cases of nontraumatic ileal perforation regardless of blood culture positivity • 1-year follow-up of blood culture–confirmed *S.*Typhi and iNTS cases and controls	• Blood samples taken from enrolled subjects at baseline • Urine samples taken from a sample of enrolled subjects at baseline • Ileal tissue samples taken in cases of nontraumatic ileal perforation regardless of blood culture positivity • 6-week phone call for blood culture–confirmed cases of *S.* Typhi or *S.* Paratyphi A—patients with complications followed up	• Blood samples taken from enrolled subjects at baseline • Ileal tissue samples taken in cases of nontraumatic ileal perforation regardless of blood culture positivity • Tier 1: Weekly follow-up, and in-person follow-up and blood collection at 28 days for enteric fever subcohort • Tier 2: Phone contact at 14 and 28 days postdischarge for cost-of-illness data	• Blood, plasma, and stool samples taken from enrolled subjects at baseline • Blood, plasma, and stool samples taken from cases and household members of culture-confirmed cases) • Day 8, 30, 180 follow-up
Incidence rate adjustment factors	• Probability of seeking care at a study facility, based on HCUS • Proportion of eligible patients enrolled in study • Proportion of eligible patients consenting to participate with a blood culture taken • Sensitivity of blood culture (assumed 60%)	• Probability of eligible patient seeking care at a study facility, based on HCUS • Proportion of eligible patients who consented and received a blood culture • Difference in healthcare-seeking according to socioeconomic status • Sensitivity of blood culture (assumed 59%)	• Probability of seeking care at a study facility, based on HCUS • Proportion of eligible patients who consented and received a blood culture • Sensitivity of blood culture (assumed 59%)	• Probability of seeking care at a study facility, based on HCUS; adjusted for the prevalence of previously identified typhoid risk factors • Proportion of eligible patients who consented and had blood drawn for culturing; adjusted for age, duration of fever, temperature at presentation, and clinical suspicion (Nepal and Bangladesh only) • Sensitivity of blood culture; adjusted for volume and reported prior antibiotic usage
Additional objectives	• Long-term sequelae, antimicrobial resistance, natural immune response, prevalence of chronic carriage, cost of illness, quality of life, long-term socioeconomic study	• Long-term sequelae, antimicrobial resistance, cost of illness	• Antimicrobial resistance • Cost of illness	• Prevalence of chronic carriage, seroincidence, antimicrobial resistance, household transmission

Abbreviations: HCUS, healthcare utilization survey; iNTS, invasive nontyphoidal Salmonella; SEAP, Surveillance for Enteric Fever in Asia Project; SEFI, Surveillance of Enteric Fever in India; SETA, Severe Typhoid Fever Surveillance in Africa; STRATAA, Strategic Typhoid Alliance Across Africa and Asia.

Each study generates both crude and adjusted incidence rates, but the approach to defining population denominators and the choice of adjustment factors used differ. Population denominators affect both crude and adjusted incidence rate calculations, so this distinction is important. The STRATAA approach to estimating the population denominator is different from the approach used by SEAP, SETA, and SEFI tier 2, as the demographic censuses provide precise population denominators. In SEAP, SETA, and SEFI tier 2, eligible cases come from predefined geographic catchment areas, which is arguably less precise but also less resource-intensive, and enables surveillance to cover larger catchment areas. Each study employs an adjustment for blood culture sensitivity, but STRATAA samples from probability distributions informed by a recent meta-analysis of blood culture sensitivity by volume of blood acquired per subject and reported prior antibiotic usage [25], whereas SEAP and SETA apply the same correction factor (assuming sensitivity of 59%) to each blood culture result [26]. For the healthcare-seeking adjustment, SETA assumes the same risk of typhoid infection for patients who seek care at a study facility and for patients who seek care elsewhere, whereas SEAP, SEFI tier 2, and STRATAA assume a differential typhoid risk for febrile patients who seek care at a study facility [13, 27]. SEFI tier 1 does not include a healthcare-seeking adjustment, since it employs active surveillance.

**Figure 1.**
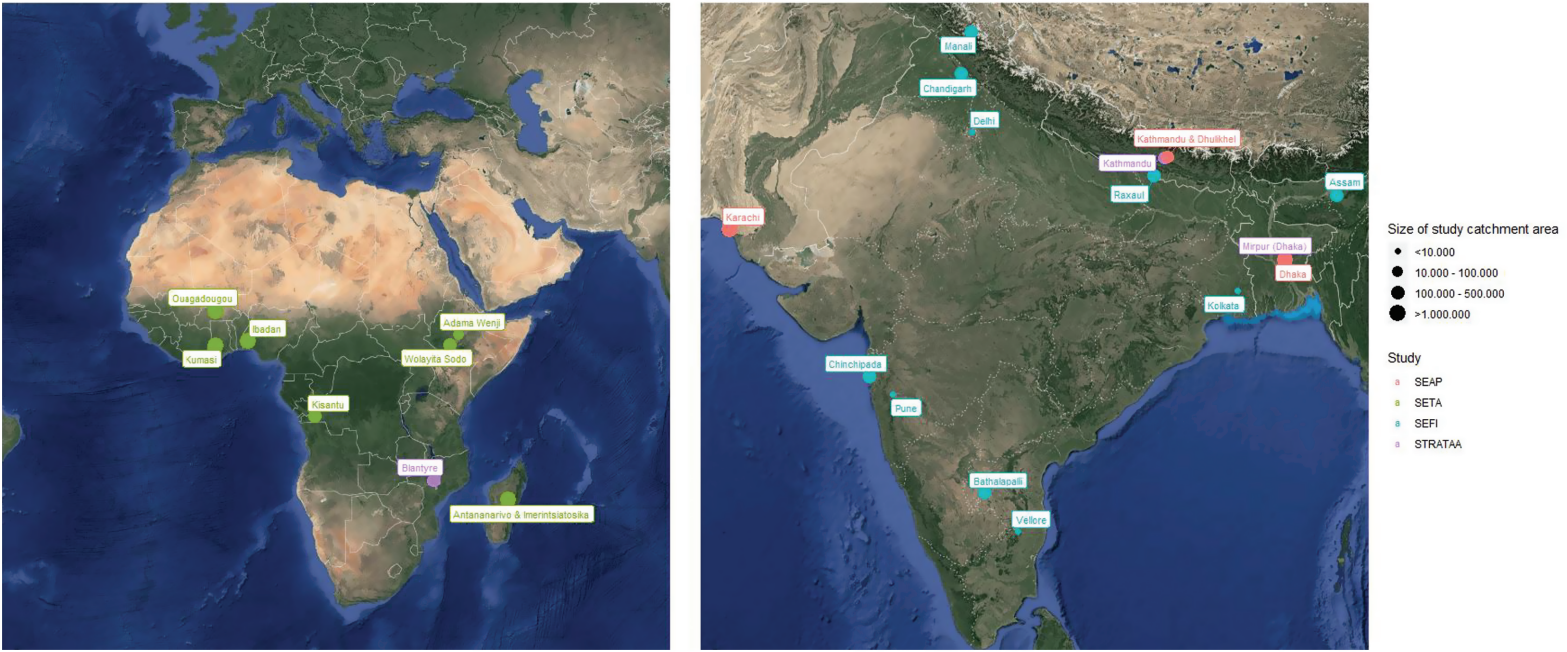
Locations and catchment area sizes for Surveillance for Enteric Fever in Asia Project (SEAP), Severe Typhoid Fever Surveillance in Africa program (SETA), Surveillance of Enteric Fever in India (SEFI), and Strategic Typhoid Alliance Across Africa and Asia (STRATAA) surveillance sites.

There are also key differences between these studies based on the geographies under surveillance, which are illustrated in Figure 1. The STRATAA sites are in densely populated, urban areas, whereas SEFI, SETA, and SEAP have a mixture of urban, periurban, and rural sites. There are observed differences in physician practices around administration of blood culture and clinical familiarity with typhoid fever among some South Asian sites, and differences in the availability of antimicrobials across all study sites. There are differences in preexisting capacities across sites to conduct routine blood culture surveillance, and the difference in blood volumes collected and antimicrobial usage across sites could lead to variability in the sensitivity of results. There is also a great deal of variability in accessibility of healthcare facilities, local water and sanitation behaviors infrastructure, and fecal sludge management.

There is value in the diversity of these study approaches. Some methodological differences are driven by rational, pragmatic choices made by study investigators that reflect differences between sites captured above. Each study addresses distinct data gaps, like potential transmission routes, prevalence of chronic carriers, and duration of immune response to natural infection, all of which will potentially inform optimal intervention strategies. Each study includes efforts to validate 1 or more low-cost alternatives to blood culture surveillance, which means that there is likely to be greater clarity in the near future about which approaches, if any, are viable, feasible, and cost-effective. The diversity of settings under surveillance broadens our understanding of the global incidence rates of typhoid fever and other invasive *Salmonella* infections, which will impact prioritization and targeting of combination vaccine development efforts. Having more broadly representative genomic data also facilitates monitoring the evolution and spread of different antimicrobial resistance genotypes, which also should inform more sophisticated targeting of interventions.

## CONCLUSIONS

In a position paper from 2008, the WHO stated that the deployment of the then-available typhoid vaccines “should be based on detailed knowledge of the local epidemiological situation” while simultaneously acknowledging the limitations of the existing data, particularly for the African continent [7]. In the intervening decade, the availability of safe, immunogenic, and efficacious TCVs [23, 28] has strengthened the need for accurate burden of disease data across diverse epidemiological settings. In this article, we have described the design and methodology of 4 landmark surveillance studies that collectively incorporate 44 surveillance facilities, 11 countries, and a total population under surveillance of > 20 million people. We argue that these studies have made large strides toward achieving the 2008 targets, and it is hoped that this article provides an important overview of the methodologies, strengths, and limitations of each individual study. Together, these data will provide key stakeholders and country-level decision makers with more accurate estimates of disease burden, allowing targeted, timely, and cost-effective deployment of new preventative strategies.

The TSAP study demonstrated that there was a significant incidence of typhoid fever and iNTS disease in Africa. SETA, SEAP, SEFI, and STRATAA are generating data on disease transmission, risk factors, cost of illness, and incidence of severe disease in Africa and some parts of Asia. Furthermore, these studies have provided baseline data in support of ongoing or planned phase 3/4 TCV trials. Data from these studies are now being used to enable national stakeholders to make informed decisions on optimal TCV delivery strategies, and to direct the development and prioritization of future *Salmonella* combination vaccine approaches. Methodological differences notwithstanding, each study has contributed important data to advance global typhoid control.

Each surveillance study will provide an estimate of disease burden for a particular setting at a particular point in time and will reflect transmission dynamics specific to a particular setting. We acknowledge that caution should be exercised when extrapolating these figures in an attempt to provide country-level estimates of disease burden, as the true distribution of disease is likely to display marked intra- and intercountry and temporal variation [29]. Furthermore, estimates of disease burden are unlikely to be static and will likely change in response to improvements or breakdown of sanitation infrastructure and the deployment of TCVs. Consideration should be given toward the establishment of ongoing surveillance programs to track changes in spatial and temporal trends in disease incidence. Such programs should be integrated into broader, vaccine-preventable disease surveillance efforts, and should potentially incorporate new, lower-cost alternatives to blood culture surveillance if and when these methods are validated, for maximal sustainability.

The updated 2018 WHO position paper on typhoid vaccination recommends that “endemic countries strengthen the surveillance of typhoid fever in all age groups, and monitor the presence of antimicrobial resistant strains of *S*. Typhi in endemic and epidemic disease, before and after introduction of typhoid vaccines” [10]. Nevertheless, the large surveillance studies described herein are unlikely to be funded in perpetuity, and a shift in emphasis may need to be made toward strengthening routine national surveillance systems with a more targeted remit. It is therefore imperative that the existing studies achieve maximal utility by addressing outstanding questions relating to age-specific incidence, transmission, carriage, strain/serovar replacement, and incidence post-TCV introduction. In addition, the continued validation of alternative low-cost methods for typhoid surveillance at these sites could yield substantial benefit to the field.

## Notes

***Financial support.*** This work was supported by the Bill & Melinda Gates Foundation (BMGF) (grant number OPP1182032).

***Supplement sponsorship.*** This supplement is funded with support from the Coalition against Typhoid Secretariat, housed at the Sabin Vaccine Institute in Washington, DC and made possible by a grant from the Bill & Melinda Gates Foundation.

***Potential conflicts of interest.*** A. J. P. is chair of the UK Department of Health’s Joint Committee on Vaccination and Immunisation and the European Medicines Agency Scientific Advisory Group on Vaccines; is a member of the World Health Organization (WHO) Strategic Advisory Group of Experts; and is a UK National Institute for Health Research (NIHR) Senior Investigator. V. E. P. is a member of the WHO Immunization and Vaccine-Related Implementation Research Advisory Committee, and reports grants from Wellcome Trust, during the conduct of the study. J. A. reports a patent pending, related to serodiagnostics for enteric fever. I. N. O. reports grants and nonfinancial support from the International Vaccine Institute, and grants from the UK Medical Research Council and Department for International Development and the NIHR, during the conduct of the study; and is a member of the BMGF Surveillance Advisors Group (from 2019 onward) and the Surveillance and Epidemiology of Drug Resistant Infections Consortium supported by the Wellcome Trust. S. S. reports grants from the National Institute of Health and Research, BMGF, WHO, Pfizer, GlaxoSmithKline, and Sanofi Pasteur, outside the submitted work. All other authors report no potential conflicts of interest. All authors have submitted the ICMJE Form for Disclosure of Potential Conflicts of Interest. Conflicts that the editors consider relevant to the content of the manuscript have been disclosed.
